# Supplementary material for: A high-resolution mRNA expression time course of embryonic development in zebrafish
Source: eLife. 2017 Nov 16;6:e30860. doi: 10.7554/eLife.30860 (PMC5690287; doi:10.7554/eLife.30860)
Supplement: Supplementary file 6. [file elife-30860-supp6.zip › biolayout-clusters-files/Cluster096-genes.html]

Cluster096


# Cluster096: Genes

| Ensembl ID | Gene Name | Chr | Start | End | Biotype |
| --- | --- | --- | --- | --- | --- |
| ENSDARG00000056233 | TMEM179B (1 of many) | 7 | 19657806 | 19671281 | protein\_coding |
| ENSDARG00000098753 | elmo1 | 19 | 34300445 | 34476377 | protein\_coding |
| ENSDARG00000022763 | hpcal1 | 20 | 31330044 | 31405926 | protein\_coding |
| ENSDARG00000042853 | ivd | 17 | 31329597 | 31343993 | protein\_coding |
| ENSDARG00000004115 | mgat4b | 14 | 16178273 | 16448609 | protein\_coding |
| ENSDARG00000043275 | mgmt | 17 | 20194377 | 20198555 | protein\_coding |
| ENSDARG00000027345 | mpzl2b | 5 | 37339568 | 37363610 | protein\_coding |
| ENSDARG00000037551 | pm20d1.1 | 23 | 18094585 | 18104060 | protein\_coding |
| ENSDARG00000016584 | rgs7a | 17 | 19606643 | 19707276 | protein\_coding |
| ENSDARG00000062330 | si:ch211-233a24.2 | 13 | 12651814 | 12853463 | protein\_coding |
| ENSDARG00000092455 | si:ch211-246e12.3 | 3 | 18484945 | 18500900 | protein\_coding |
| ENSDARG00000031302 | smap1 | 13 | 27879277 | 28133418 | protein\_coding |
| ENSDARG00000010981 | zdhhc3a | 16 | 39246695 | 39283419 | protein\_coding |
